# An Adenoviral Vector Based Vaccine for *Rhodococcus equi*

**DOI:** 10.1371/journal.pone.0152149

**Published:** 2016-03-23

**Authors:** Carla Giles, Olasumbo Ndi, Mary D. Barton, Thiru Vanniasinkam

**Affiliations:** 1 School of Pharmacy and Medical Sciences, University of South Australia, Adelaide, South Australia, Australia; 2 School of Biomedical Sciences, Charles Sturt University, Wagga Wagga, New South Wales, Australia; Ross University School of Veterinary Medicine, SAINT KITTS AND NEVIS

## Abstract

*Rhodococcus equi* is a respiratory pathogen which primarily infects foals and is endemic on farms around the world with 50% mortality and 80% morbidity in affected foals. Unless detected early and treated appropriately the disease can be fatal. Currently, there is no vaccine available to prevent this disease. For decades researchers have endeavoured to develop an effective vaccine to no avail. In this study a novel human adenoviral vector vaccine for *R*. *equi* was developed and tested in the mouse model. This vaccine generated a strong antibody and cytokine response and clearance of *R*. *equi* was demonstrated following challenge. These results show that this vaccine could potentially be developed further for use as a vaccine to prevent *R*. *equi* disease in foals.

## Introduction

*Rhodococcus equi (Prescottella equi*) [1,2], is a Gram positive, aerobic coccobacillus which primarily infects foals under 6 months of age, causing suppurative bronchopneumonia and pyogranulomatous lesions [3–5]. *R*. *equi* is also known to infect pigs [6] and immunocompromised humans[7]. This bacterium is a common soil saprophyte, has a worldwide distribution and causes an estimated 3% of all foal deaths and has a mortality rate of approximately 50%[8,9]. Virulence and intracellular survival of *R*. *equi* within the macrophage is regulated by the plasmid bound virulence associated proteins (Vaps) designated A-I and X which are located within a pathogenicity island[10]. VapA is the primary protein involved in the bacterial pathogenicity and virulence and is further regulated by the *virR* and *orf8* genes[11,12].

Current treatment for *R*. *equi* infection consists of combination antimicrobial therapy using rifampin and macrolides[13]; however, recent reports of resistance in the USA [14]and China [15] indicate that the need for prevention rather than reliance on treatment of this infection is becoming critical. For decades researchers have been attempting to find an effective vaccine for use in foals that is safe, immunogenic and efficacious[16]. However, understanding of the foal immune system is limited and this has slowed vaccine development [17] although recent findings have demonstrated the importance of both humoral and cell mediated immune responses to effectively combat *R*. *equi* infection[18,19]. By approximately 3–4 months of age the foal is capable of producing an immune response sufficient against *R*. *equi*[20, 21], making a vaccine that is effective in early life imperative.

*R*. *equi* vaccine candidates based upon the traditional vaccine platforms such as live[22], killed [23, 24] and attenuated [25] have not been successful. Modern molecular based vaccines for example DNA[26, 27], subunit [28] and genetically attenuated *R*. *equi*[29, 30], have shown some potential, however, these vaccines have not conferred protection. More recently bacterial vector vaccines utilising the *vapA* gene have been tested and have shown some promise [31,32] but are yet to be developed further.

Adenoviral vector vaccines have been shown as an effective vaccine modality capable of generating strong CD8^+^ T cell and B cell responses with low pathogenicity and are safe and stable[33]. Notably, the adenoviral vector has proven itself to be a suitable candidate for use in infant mammals such as puppies[34], piglets [35] and mice [36, 37]. Amongst the *R*. *equi* related bacteria, *Mycobacterium tuberculosis* is one example of an intracellular pathogen for which the adenoviral vector platform has been used successfully and one such vaccine is currently being tested in clinical trials in human infants[38]. In this study an adenoviral vector vaccine based upon the human adenovirus serotype 5 (HAdV5) containing the *R*. *equi vapA* gene [23, 39] was developed and tested in mice for safety, immunogenicity and efficacy.

## Materials and Methods

### Vaccine construct

The AdenoX HAdV-vapA vaccine construct, utilised a HAdV5 vector (Clontech, USA) and the *R*. *equi vapA* gene was inserted into the viral construct using the methods described by the manufacturer. VapA primers were forward 5’ gtaactataacggtcatgaagactcttcacaagacggt 3’ and reverse 5’ attacctctttctccctaggcgttgtgccagcta 3’ with the tagged sequence for vector insertion underlined. The product was 570bp in size and was confirmed via Sequencing (Flinders sequencing, Adelaide, Australia) for ligation. HAdV-*vap*A Virus was grown on HEK 293 cells, with cells maintained on complete DMEM (Life Sciences, Australia), 10% Foetal Bovine Serum (Sigma-Aldrich, Australia), 2% L-glutamine (Life Sciences, Australia) and 2% penicillin/streptomycin (Life Sciences, Australia) The developed HAdV-*vapA* was verified for transcription and translation by Reverse transcriptase-PCR and Western Blot using anti-rabbit VapA polyclonal antibody (dilution 1:5000) (SAHMRI, Adelaide, Australia). Virus was purified and quantitated using the AdEasy virus purification and titration kit (Agilent, USA).

### Animals, experiments and design

All animal work conducted was approved by the SA Pathology Animal Ethics Committee, approval numbers 187a/12 and 72b/13. Female C3H/HeJ (Animal resource Centre, Perth, Australia), aged 6–8 weeks were distributed randomly into individual ventilation cages (n = 3–5). Animals were monitored daily throughout the trial and after challenge animals were monitored twice daily. Clinical records sheets were maintained and animal health was recorded and scaled according to different clinical signs, if the total score was equal to or over 4 mice were to be euthanised immediately. Of the mice involved in this trial only one unexpected death was experienced and upon post mortem by a veterinarian, the cause of death was due to cancerous tissue in the lung. Stress was minimised in this trial by ensuring animals were first grouped with other mice of similar sizes to minimise bullying; handling was kept to a minimum with mice weighed weekly and given fresh food water and cages at this time and bleeding and vaccination by intramuscular (IM) injection occurred every other week at the time of weighing. Cheek vein bleeding was used in order to avoid the use of further anaesthetics required for other venipuncture methods. Cheek vein junction bleeding allows for clotting to occur as soon as the skin is released. If at any time the mice displayed excessive stress the bleeding was ceased and animals placed into their new cage and the experience was recorded on the clinical records sheet. Animals were humanely killed by first undergoing anaesthesia by isofluorane, cardiac puncture and cervical dislocation before organs were harvested for culture.

Mice were bled by the cheek vein junction in trial 1 at days 0, 14, 28, 42 and 49 or trial 2 at endpoint days 31, 33 or 35. Mice were vaccinated at day 0 and 14, and challenged at day 28 by aerosol with 1x10^9^ CFU/ml of ATCC 33701 *R*. *equi*.

Two sets of studies were conducted; the first contained four vaccine regimes of 6 mice in each. The vaccines administered were administered by IM injection, 50 μl of vaccine administered into each quadriceps muscle group with a total dose of 100 μl per mouse. The vaccines administered were Live *R*. *equi* ATCC 33701 (100μl of 1x10^5^ CFU/ml) in a prime dose only, empty vector HAdV (100μl of 1x10^9^ ifu/ml) prime dose only, HAdV-*vapA* (100μl of 1x10^9^ ifu/ml) prime dose only and HAdV-*vapA* (100μl of 1x10^9^ ifu/ml) in a prime/boost regime 14 days apart,

The second trial again consisted of four vaccine regimes all administered in a prime/boost regime 14 days apart; Live *R*. *equi* ATCC 33701 (100μl of 1x10^6^ CFU/ml), empty vector HAdV (100μl of 1x10^9^ ifu/ml) prime only; HAdV-*vapA* high dose (100μl of 1x10^10^ ifu/ml) and HAdV-*vapA* low dose (100μl of 1x10^9^ ifu/ml) again administered by IM.

### Enzyme Linked Immunosorbent Assay (ELISA) to detect VapA total IgG and IgG subclasses

ELISA to detect VapA specific total IgG and IgG subclasses (IgG2a, IgG2b, Ig1) were conducted using standard protocols and all wash steps were performed 3 times. Briefly, the plate (Nunc Maxisorp, Nunc, USA) was first coated in a VapA protein extraction that was undertaken according to previously published protocols utilising the Triton X 114 phase partitioning *R*. *equi* based protocol[40, 41]. Plate wells were coated with 100 μl/well of 3.5 μg/ml APTX VapA antigen in a calcium carbonate coating buffer and incubated overnight at 4°C. Followed by a washing step, whereby contents was decanted, wells were each filled with approximately 200μl of washing buffer (PBS/Tween 20, 0.05% v/v), and again decanted and plates tapped onto absorbent towel to remove residue and repeated 3 times. The plates were then blocked with 150 μl/well of blocking buffer 1% bovine serum albumin (BSA) (Sigma-Aldrich) in PBS and incubated at room temperature for 1 hour. Plate was washed, and serum samples, diluted 1:100–1:1600 in a 2 fold fashion were added at 100 μl/well and incubated at 37°C for 1 hour. Again plates were washed, and 100 μl/well of anti-mouse HRP antibody at and plates were incubated for 1 hour at room temperature. Secondary antibody concentrations were Total IgG- 1:10 000 (Sigma, Australia), IgG1- 1:5 000 (Genetex, USA), IgG2a- 1:5000 (Genetex, USA) and IgG2b- 1:5000 (Genetex, USA). Plates were again washed and 100 μl/well of TMB—3, 3’,5, 5’-Tetramethylbenzidine in 0.05% citrate acetate buffer (Sigma-Aldrich) and activated by 30% hydrogen peroxide was added to each well and incubated in the dark, at room temperature for 15 min. Finally, the reaction was stopped with 1 M Hydrochloric acid (HCl) and absorbance read at 450 nm.

Empty Vector ELISA was conducted with the same protocol as the VapA total IgG ELISA with the antigen being a 100 μl/well of 1x10^5^ ifu/ml HAdV empty vector virus, inactivated by heat inactivation at 56°C for 30 minutes.

Total VapA IgG serum levels were tested in trial 1 and 2 utilising murine serum pooled per group which were: *R*. *equi* prime only, HAdV empty vector prime only, the developed HAdV-*vap*A vaccine vector prime only and the HAdV-*vap*A prime and boost.

### Mouse lung cell culture

Isolation of lung cells was performed as described by [28, 42] with minor modifications with cytokine analysis only performed on trial 2 mice. This was due to the background levels of cytokines present in trial 1 at day 49 (5 weeks post challenge). The pooled lungs for each vaccine group according to their euthanasia day (n = 6 day 35) were diced and the supernatant discarded and the tissue suspended in 3 ml of RPMI 1640 media containing 1 mg/ml Collagenase XI (Sigma-Aldrich) and 30 μg/ml DNAse (Sigma-Aldrich) and placed in 10 ml tube shaken at 37°C for 45 min. Supernatant was collected and 7 ml of sterile PBS was added and the tube was centrifuged at 200 g for 10 min. The supernatant was discarded and the cells resuspended in 2.5 ml of complete RPMI media (Sigma-Aldrich, Australia). A concentration of 1 ml of 1x10^6^ cells/ml in complete RPMI media or with the stimulant ConA were cultured in triplicate on 24 well plates, with 1ml of cells per well and incubated at 37°C, 5% CO_2_ for 5 days, cell culture supernatant was collected and stored at -20°C until assayed for cytokine levels.

### Cytokine detection from stimulated lung cells

The Luminex xMAP system was used to detect presence of cytokines- IL-2, IL-4, IL-10 and IFN-γ from both stimulated lung cell culture assays using the Milliplex Map Kit (Millipore, Australia) and the procedure was completed following the manufacturer’s directions and read on the Luminex 200, HTS, FLXMAP 3D MAGPIX with xPONENT software. Samples were prepared according to the requirements of the kit. Cell culture supernatant samples were pooled according to vaccine treatment group and centrifuged to remove any cell debris and particulate matter.

### Challenge studies

Mice were infected with 1x10^9^ CFU/ml of aerosolised *R*. *equi* using a nebuliser pump therapy kit (Ventalair forte II, Allersearch, Australia) and the protocol was conducted as described by Phumoonna *et al*., (2005). Briefly, the entire cage of mice (5 or 4 depending on the cage), was placed for 20 min in a sealed plastic chamber which measured 265 mm x 235 mm x 120 mm and had a removable sealed lid for ease of access. The 3 ml bacterial suspension was aerosolised using a nebuliser kit (Ventalair forte II), an inlet port on the bottom of the chamber allowed for attachment of the nebuliser bowl inside the chamber and allowed for the attachment to the pump outside of the chamber[28]. Due to the positive pressure the nebuliser pump generated there was a small sealed outlet port on the side of the chamber, connecting to a hose which in turn was connected to a stoppered flask containing 70% (v/v) ethanol as a safety measure, to ensure no bacteria leaked into the environment. Mice were placed in the chamber for a total of twenty minutes with the nebuliser pump on for 15 min to allow for the nebuliser bowl to be emptied and then for a further 5 min with the nebuliser pump switched off to ensure mice received a full dose of *R*. *equi*.

Immediately after the 20 min aerosol *R*. *equi* challenge, one randomly selected mouse was euthanised from each treatment. One lung from each mouse was harvested for bacterial culture where the lung was first weighed and then homogenised with scalpel blades in 500 μl of saline and vigorously mixed. The suspension was then serially diluted 10 fold (10^0^–10^9^) in saline and in duplicate 100 μl of suspension were plated onto M3T media [43, 44] to select for *R*. *equi*. Plates were incubated for 48 hr at 37°C and then counted. Bacterial counts were calculated in CFU/g to allow for a more accurate determination of the retained bacterial dose.

### Histological studies

Histological sections of lungs were preserved in formalin buffer and sectioned in paraffin wax. Slides were stained with Haematoxylin and Eosin, and Grams tissue stain using standard techniques described[45]. Lung sections were examined by a veterinary pathologist. Both haematoxylin and eosin (H & E), and Gram stained sections showed the effects of the challenge on the lung and the presence of *R*. *equi* respectively, The H & E stain was scored on a scale of 0–10 with 0 = no changes (normal pathology) and 10 = multiple granulomata in every field.

### Western Blot to detect VapA expression

Western blots were conducted utilizing standard methods [46] with the primary antibody used a rabbit anti-VapA polyclonal antibody (SAMHRI, Australia) at 1:500. Secondary antibody 1:5000 anti-rabbit HRP (Sigma-Aldrich, Australia) imaged immediately on the ImageQuant LAS 4000 (GE Healthcare Life Sciences).

### Detection of HAdV vector in mouse faeces

RNA extraction of mouse faeces to detect HAdV was undertaken according to the protocol of the viral RNA extraction kit (Invitrogen). Reverse transcriptase PCR (RT-PCR) was undertaken using the kit and protocol of the ProtoScript^®^ Taq RT-PCR kit (New England Biolabs). PCR amplification of the cDNA was then performed with using *vapA* PCR primers *vapA* forward, 5’ gtaactataacggtcatgaagactcttcacaagacggt 3’; *vapA* reverse 5’ attacctctttctccctaggcgttgtgccagcta 3’.

### Statistical analysis

Statistical analysis was generated by the GraphPad Prism 6 for Windows (GraphPad Software, La Jolla California USA, www.graphpad.com); tests undertaken, were the t test unpaired analysis with 95% confidence.

## Results

### Trial 1

Trial 1 was undertaken to determine the safety and immunogenicity of the newly developed HAdV-*vapA* vaccine. Mouse faecal samples were collected and reverse transcriptase PCR (RT-PCR) was performed on these samples to determine if virus was shed by the mice following vaccination.

#### VapA specific antibody response

**Fig 1:**
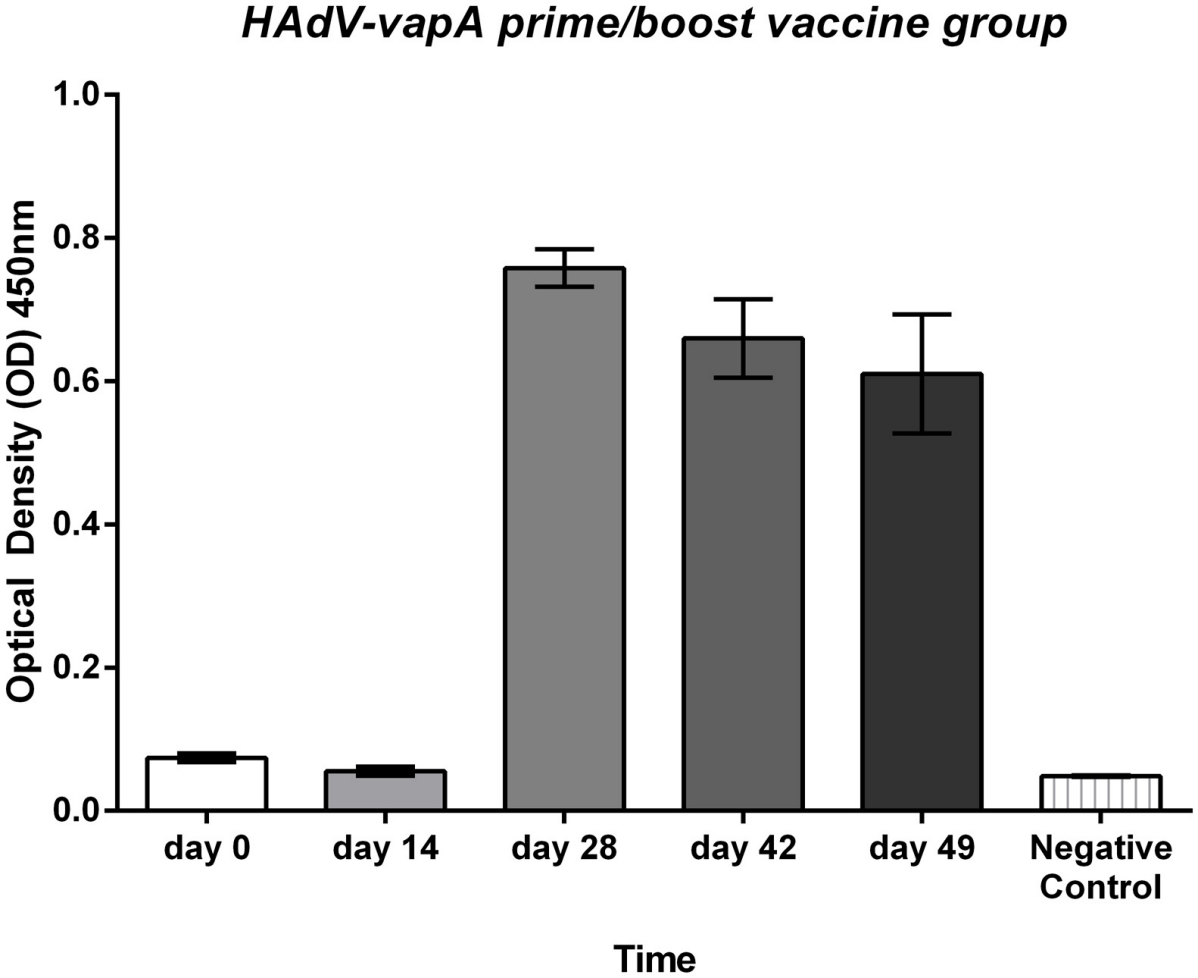
Trial 1 HAdV-*vapA* prime/Boost regime VapA total IgG ELISA response. Mean and SD VapA total IgG antibody levels at a 1:100 dilution of mouse sera pooled in the HAdV-*vapA* prime/boost vaccine regime per group over the duration of trial 1. Sera was collected at day 0 pre vaccination; day 14, 2 weeks post prime; day 28, 2 weeks post boost; day 42, 4 weeks post boost; and day 49 5 weeks post boost.

The HAdV-*vapA* prime boost elicited a strong VapA specific IgG response (Fig 1), while the empty vector HAdV vaccine regime did not produce a VapA specific antibody response. The *R*. *equi* vaccine did not produce a detectable total VapA specific IgG antibody response. The dose rate 1x10^5^ CFU/ml of live virulent *R*. *equi* may have been cleared too rapidly from the mice to enable generation of an antibody response.

The HAdV-*vapA* prime only did not elicit a total IgG antibody response that was detectable. However, the HAdV-*vapA* prime and boost regime produced a strong VapA specific total IgG response, with these levels sustained until day 49 (5 weeks post boost).

### Trial 2

Trial 2 was conducted to determine the efficacy of the HAdV-*vapA* vaccine and further validate the immunogenicity of this vaccine.

#### VapA specific antibody responses

**Fig 2:**
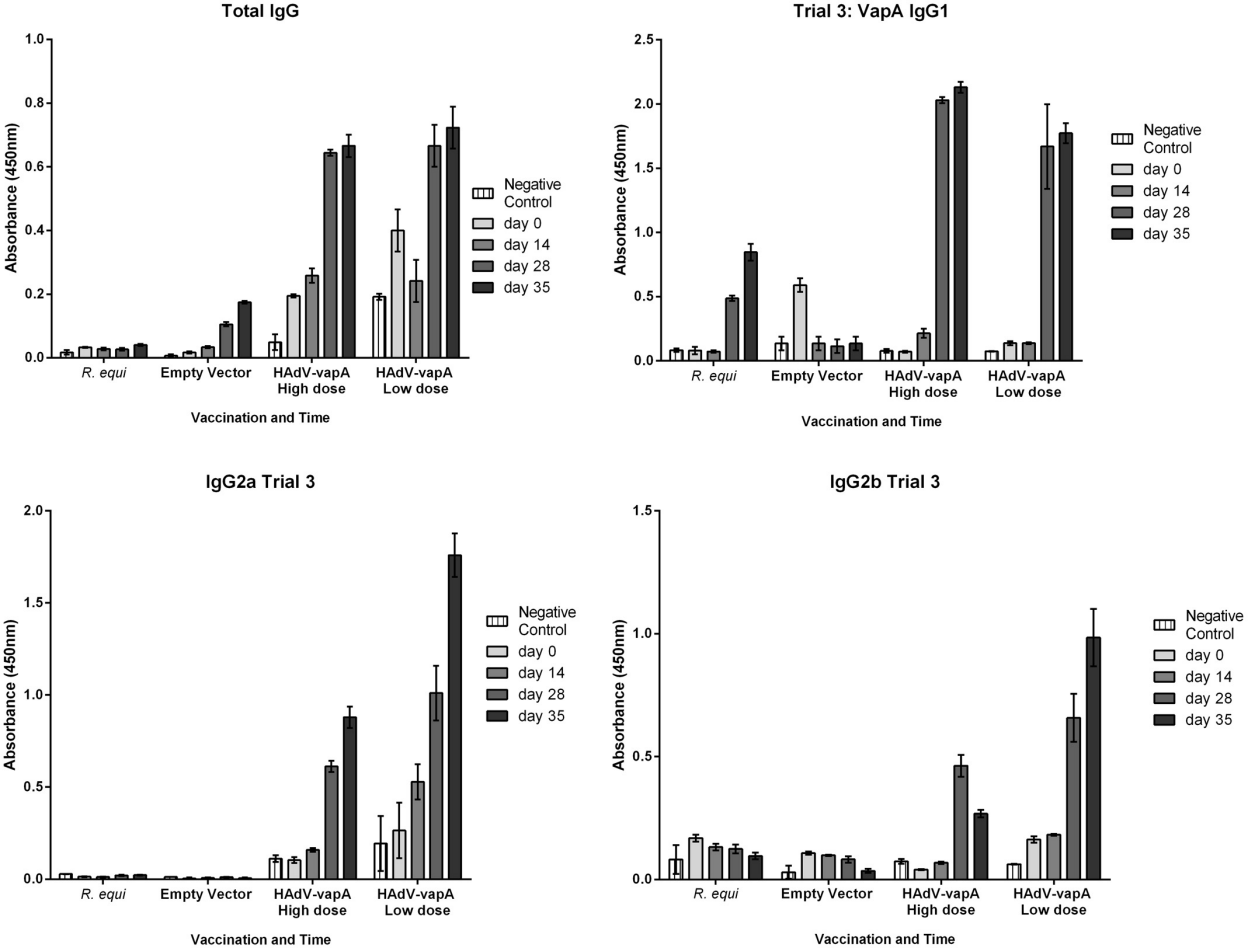
Trial 2 VapA specific antibody responses. Mean and SD of VapA total IgG triplicate OD values at a 1:100 dilution of mouse sera. A representation of time at which sera was collected at day 0 (pre vaccination), day 14 (boost vaccination), day 28 (pre challenge) and day 35 (1 week post challenge, pre euthanasia) of each vaccine group; *R*. *equi*, Empty vector negative control, HAdV-*vapA* high dose and HAdV-*vapA* low dose. Top left total IgG antibody, top right IgG1 antibody, bottom left IgG2a antibody, bottom right IgG2b antibody.

IgG responses to VapA were not seen in the empty vector or the *R*. *equi* vaccine groups (Fig 2). However, both HAdV-*vapA* prime and boost (low and high dose treatments) stimulated a high antibody response at day 28 pre challenge and a slightly higher ELISA absorbency at day 35, 7 days post challenge.

VapA specific IgG1 responses were seen in the *R*. *equi* prime/boost positive control group, with a strong IgG1 antibody response at day 28 and a potent antibody response at day 35 was seen. The HAdV-*vapA* prime/boost high dose regime elicited a stronger IgG1 response than that seen in the *R*. *equi* vaccinated positive control mice at days 28 and 35, with a slightly higher IgG1 response post challenge at titre 100 (Fig 2).

VapA specific IgG2b antibody response was not generated in the *R*. *equi* positive control or the empty vector negative control vaccinated groups. Both the HAdV-*vapA* prime/boost high and low dose regimes generated a significant IgG2b response (Fig 2).

VapA specific IgG2a antibody (Fig 2) was detected in both HAdV-*vapA* low and high dose vaccinated groups, with a potent antibody response seen at days 28 and 35 post vaccination. While the *R*. *equi* prime and boost vaccinated group and the empty vector prime and boost vaccinated mice did not demonstrate a significant VapA specific IgG2a responses and this outcome is consistent with other results from this trial.

#### HAdV capsid specific total IgG response

**Fig 3:**
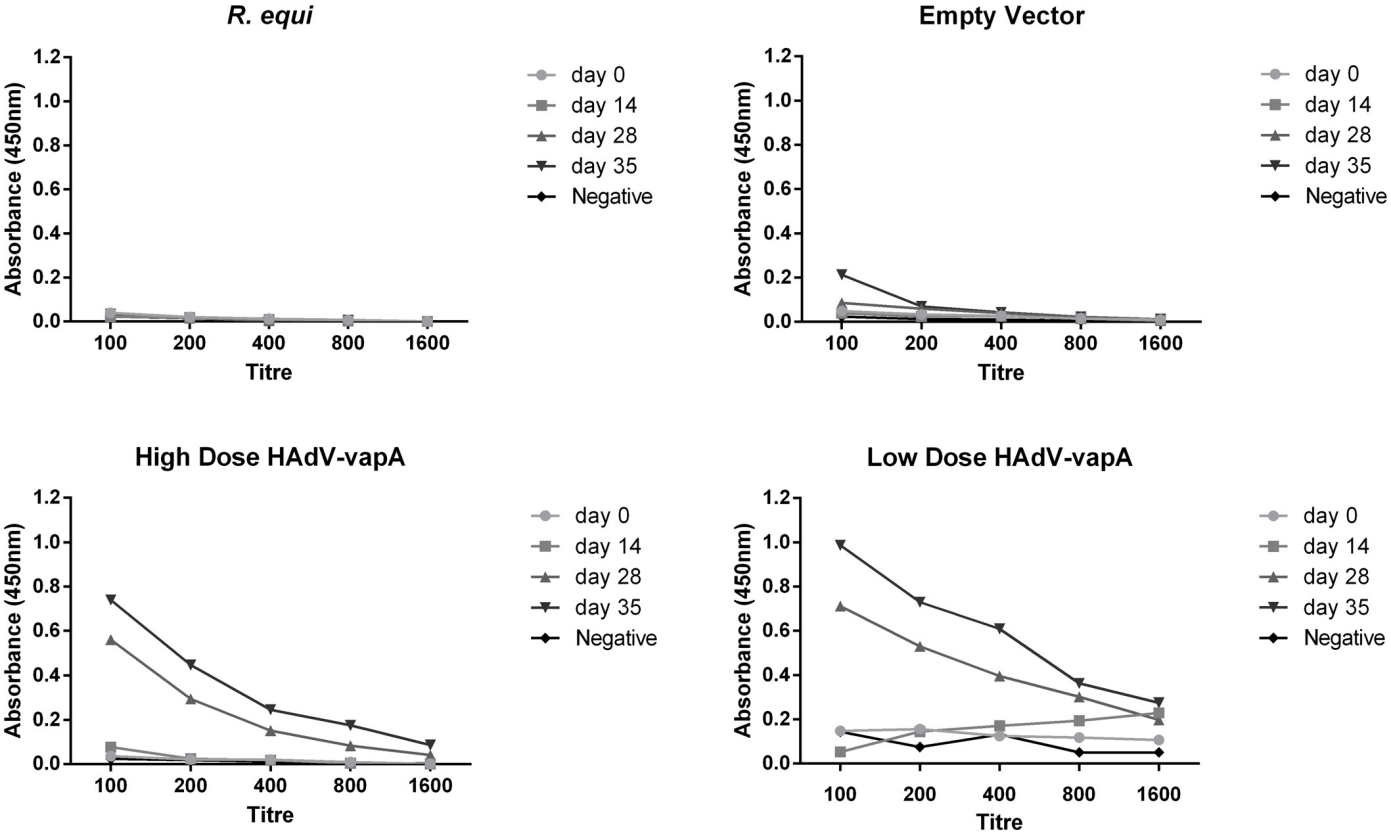
HAdV Capsid specific Total IgG antibody response in mice (tested at different time points) and titre. Diluted HAdV capsid antibody levels over the 35 day duration of the trial. Mouse sera pooled per vaccine group, diluted in a 2 fold fashion from 100–1600. Sera was collected at day 0 (pre vaccination), prime vaccine administered, day 14 (boost vaccination), day 28 (pre challenge) and day 35 (1 week post challenge, pre euthanasia) of each vaccine group; *R*. *equi* positive control, Empty vector negative control, HAdV-*vapA* high dose and HAdV-*vapA* low dose.

Total IgG responses to the HAdV capsid showed that there were no detectable levels of IgG antibodies present in the *R*. *equi* vaccinated groups and a strong antibody response to the HAdV-*vapA* prime/boost regimes. The empty vector produced low level of IgG response which was considered weakly positive (ELISA OD obtained was 3 times higher than the assay negative control) (Fig 3). The reason for this in unclear but this result was not seen in any of the other trials conducted with all other results showing a negative VapA specific IgG antibody response to the vector alone.

#### Cytokine response in mouse lung samples

**Fig 4:**
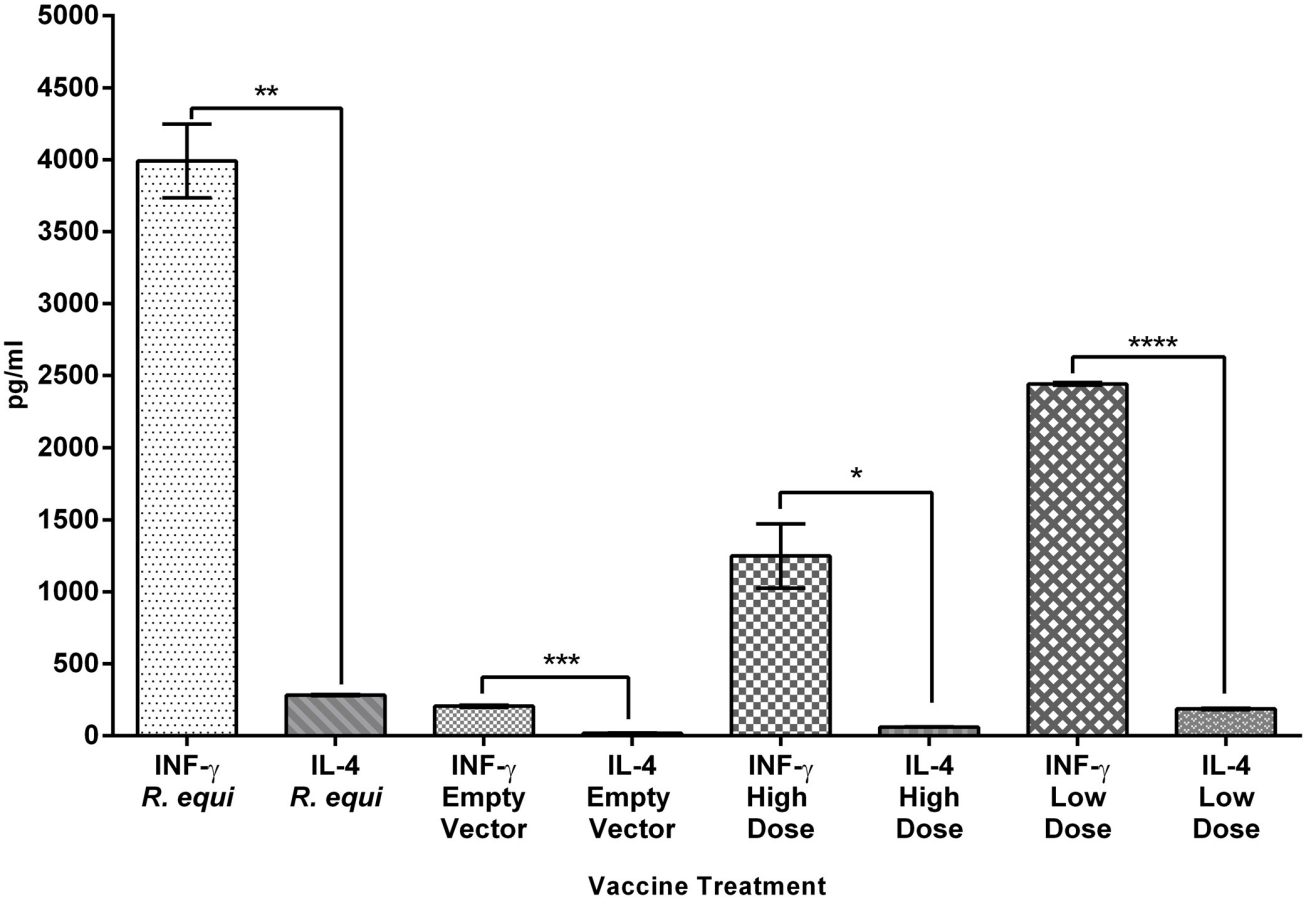
Comparison of lung INF-γ and IL-4 ConA treated cells at and day 35 collection. The mean and SD of INF-γ and IL-4 cytokines produced by lung cells from the four groups; *R*. *equi*, empty vector, HAdV-*vapA* high dose (High dose) and HAdV-*vapA* low dose (Low dose). Cytokine response was determined in culture collected at day 35 post vaccination (7 days post challenge), and were subsequently cultured and stimulated with ConA for 5 days which are represented across the X axis. Statistical analysis by the unpaired t test demonstrated significance when INF-γ to IL-4 were analysed within a treatment *P = 0.0172, **P = 0.0024, ***P = 0.0009, **** P<0.0001

**Fig 5:**
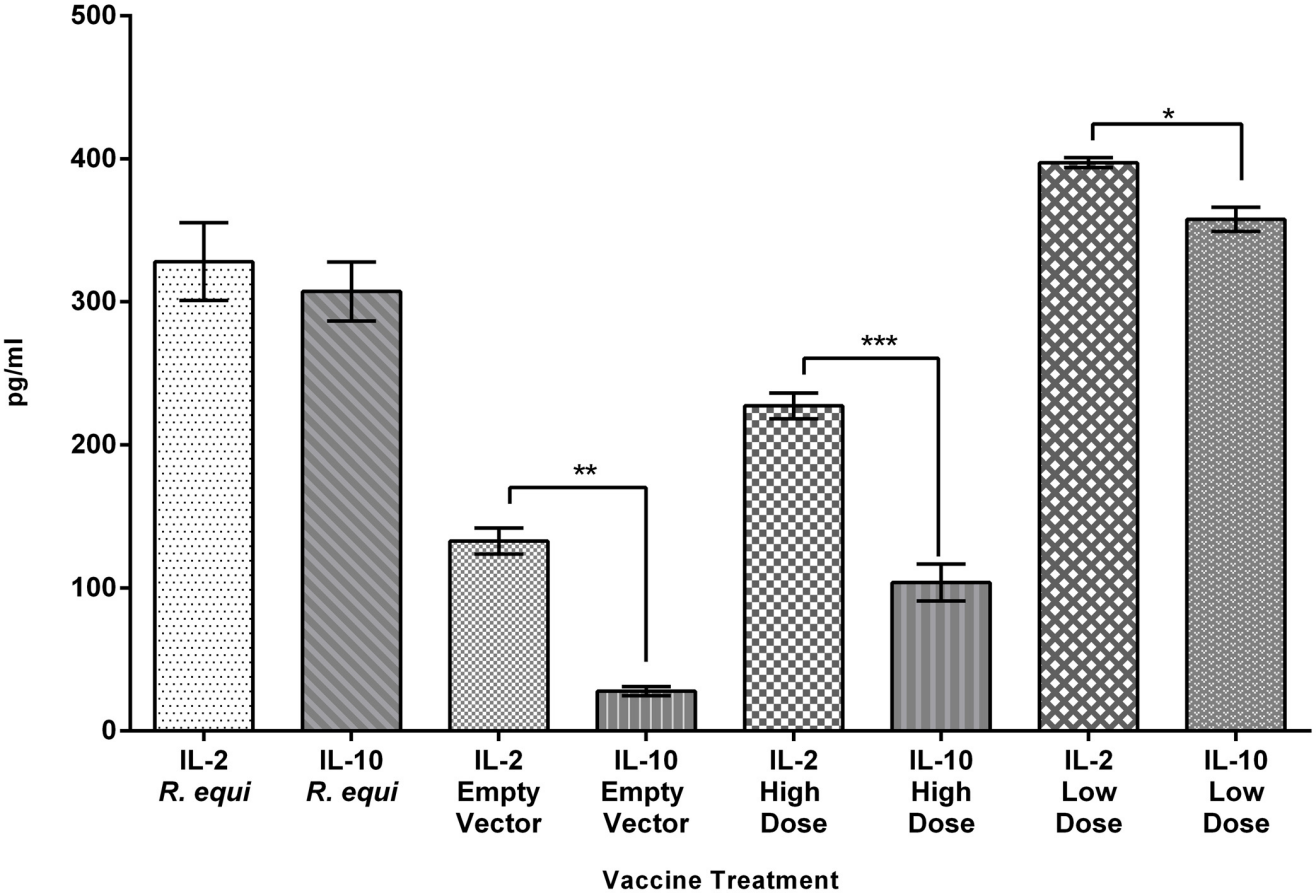
Comparison of lung IL-2 andIL-10 ConA stimulated cells collected at day 35. The mean and SD of IL-2 and IL-10 cytokines produced by lung cells from the four groups; *R*. *equi*, empty vector, HAdV-*vapA* high dose (High dose) and HAdV-*vapA* low dose (Low dose). Cytokine response was determined in culture collected at day 35 post vaccination (7 days post challenge), and were subsequently cultured and stimulated with ConA for 5 days which are represented across the X axis. Statistical analysis by the unpaired t test demonstrated in the HAdV-vapA high dose ***P = 0.0080, HAdV-vapA low dose, *P = 0.0254 and empty vector **P = 0.0042 vaccinated groups.

A mixed Th1/Th2 response was seen in the cytokines tested (Figs 4 and 5) from mouse lung cell culture supernatant that was stimulated with ConA. There was an extremely high IFN-γ response in the live *R*. *equi*, HAdV-*vapA* high and low dose vaccinated mice at day 35, while the empty vector negative controls gave lower levels of IFN-γ. The compared responses of IL-4 and INF-γ demonstrates IFN-γ has a stronger response and are significantly higher than the IL-4. This indicates a bias towards a Th1 type response which was further supported by the stronger IL2 response than IL-10 response (P = 0.0001) in HAdV-vapA vaccine groups (Fig 5).

#### Bacterial clearance Trial 2

**Fig 6:**
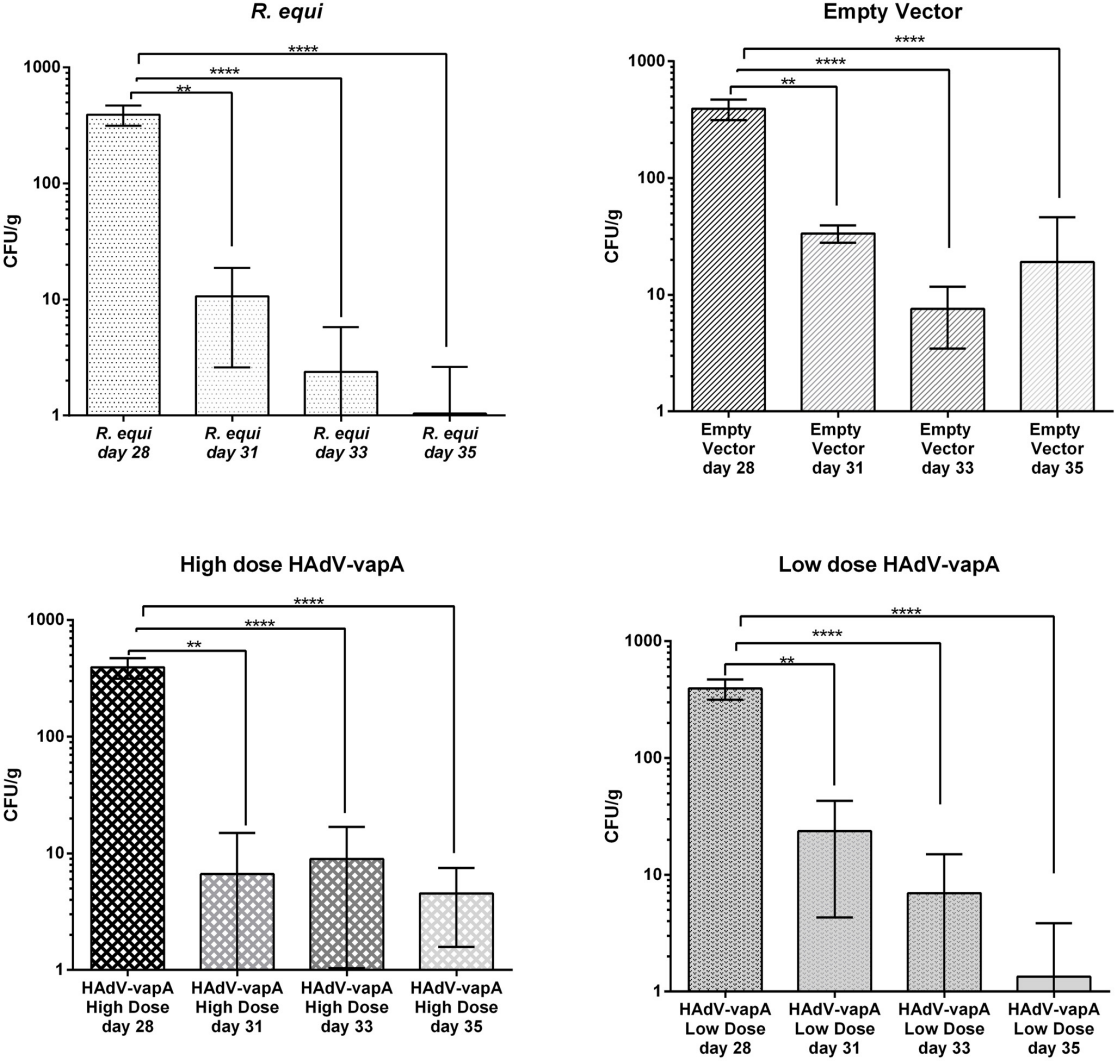
Bacterial clearance of *R*. *equi* in each treatment (Mean and SD). Mean and SD of *R*. *equi* present in the lungs (CFU/g) of mice challenged with aerosolised *R*. *equi* (1 × 10^9^CFU/ml). Collected at euthanasia at day 28 (20 min post challenge), day 31 (3 days post challenge), day 33 (5 days post challenge) and day 35 (7 days post challenge) from the 4 vaccine groups; *R*. *equi (top left)*, empty vector (top right), HAdV-*vapA* high dose (bottom left) and HAdV-*vapA* low dose (bottom right). A lung was harvested, weighed and ground and *R*. *equi* cultured in 10 fold dilutions. *R*. *equi* clearance from murine lungs pooled according to vaccine group. **** p<0.0001, **p = 0.003.

Bacterial clearance was difficult to monitor, and *R*. *equi* was rapidly cleared from the mice following aerosolised challenge. As used by others [28, 47] multiple end points (days 28, 31, 33 and 35) were used to monitor the clearance of the R. equi from the mouse and to determine if any histological difference was seen over this period of time. As Fig 6 indicates a minor enhancement in clearance was seen between HAdV-*vapA* vaccinated groups and the empty vector vaccine group. No significance between the negative control (empty vector vaccine group) and the treated groups was seen (p>0.05). However, comparison within the treatment groups over time demonstrates significance (Fig 6).

#### Histological findings

Lung sections were examined by a veterinary pathologist. Both haematoxylin and eosin (H & E), and Gram stained sections showed the effects of the challenge on the lung and the presence of *R*. *equi* respectively, The H & E stain was scored on a scale of 0–10 with 0 = no changes (normal pathology) and 10 = multiple granulomata in every field.

Granulomata, cellular aggregations indicating resolving lesions or mild increased interstitial cellularity were detected in 4/13 mice in each of the positive control *R*. *equi* vaccination groups, the high and low dose HAdV-vapA vaccine groups, while the 6/14 negative control mice demonstrated granulomas or cellular aggregations. The presence of granulomas does support the validity of the mouse model, particularly the C3H/HeJ model for *R*. *equi* trials. The resolving nature of many of these granulomas also indicates the presence of an effective immune response to the *R*. *equi*, whether the vaccines tested in study contributed this recovery is unclear and further investigation is required to determine this. The gram stained sections showed relatively low numbers of bacteria in the lung tissues; however, this may have been due to bacteria already being cleared by the time the mice were humanely killed in this study. Two thirds (34/51) of the samples showed the presence of R. equi; some of these were engulfed within macrophage cells and some bacteria were in the interstitial spaces.

## Discussion

The studies in trials 1 and 2 demonstrated that the HAdV-*vapA* vaccine is capable of inducing a significant immune response when used in a homologous vaccine regime. A strong total IgG response and a mixed IgG subclass response to VapA, with a Th1 bias were observed. In addition, clearance of *R*. *equi* was seen to be more rapid in the lung of mice vaccinated with the HAdV-*vapA* vaccine.

A strong VapA IgG antibody response was seen in both the low and high dose HAdV-*vapA* vaccinated groups. This strong response was also maintained with high antibody levels at day 49, (5 weeks post boost), which is potentially indicative of a longer term antibody response being generated. An increase in VapA specific antibody post challenge has been seen in *R*. *equi* vaccine studies conducted in mice elsewhere[26, 48].

The lack of VapA antibody response in the *R*. *equi* positive control vaccine group in both trials 1 and trial 2 was somewhat surprising; it is possible that the antibody levels in these mice were below the detection limit of the ELISA used. The dose used here was 1 x10^5^ CFU/ml of fresh *R*. *equi* ATCC 33701, a dose that has been used elsewhere. Takai and co-workers used ATCC 33701 at 1x10^6^ CFU/ml by intravenous administration and demonstrated rapid clearance by the liver by 4 days post vaccination and an ELISA antibody OD of 1.8 at 16 days post vaccination at a sera dilution of 1:100[23]. Whereas another study by this group using mice vaccinated intravenously with 1x10^5^ CFU/ml *R*. *equi* ATCC 33701 demonstrated a strong antibody response in *R*. *equi* vaccinated mice, 5 days post vaccination[24]. This lack of antibody response seen here may be due to the rapid clearance of *R*. *equi* by the innate immune system before detectable antibody levels could be generated.

The HAdV whole capsid IgG antibody response was strong in the HAdV-*vapA* vaccinated mice while the negative control, empty vector vaccine group did not demonstrate an antibody response. This suggests that the HAdV vector itself is not highly immunogenic, which could be an advantage if this vector is to be used in a vaccine as it is not recognised by the immune system when administered without antigen, in this case VapA.

The cytokine data indicated a Th1 bias in the immune response to the HAdV vaccine and it is an approach used by other investigators[31, 32, 49]. It should be noted that a mixed Th1/Th2 immune response is present in *R*. *equi* infections in foals. A Th1 bias produced the most desirable immune response [50, 51] for the prevention of rhodococcal pneumonia in foals which typically have a Th2 predominating immune system in their early life which limits the Th1 intracellular microbicidal cytokines from being present, thus allowing *R*. *equi* to persist [52–55]. The IgG isotypes and cytokine data were useful in determining Th1/Th2 bias of the immune response to the vaccines. In future studies it would also be useful to include T cell assays to support the IgG isotype and cytokine data.

Bacterial clearance is difficult to monitor in the mouse due to the rapid natural clearance of *R*. *equi* from the mouse lung following challenge Upon *R*. *equi* aerosol challenge the vaccinated treatment groups showed some enhanced clearance, however, the difference between HAdV-*vapA* vaccinated groups and the negative control empty vector vaccine group was not significant. This is likely due to the natural rapid clearance of the *R*. *equi* from the mouse that has been observed by others[23].

A heterologous vaccine regime (HAdV-*vapA* and DNA-*vapA* as prime or boost), may be a useful to investigate in future studies as it may improve the efficacy of the vaccine developed in this study. Previous studies have shown DNA and adenoviral vector vaccines-based heterologous vaccine regimens to be effective against other intracellular bacterial pathogens such as *Mycobacterium tuberculosis*[56, 57]. Generally, researchers advocate the use of mixed modality vaccine regimes as there is reduced risk of the development of neutralising antibodies to the vector as could potentially occur when homologous vaccines are used[33]. In future studies other immunogenic antigens such as GroEL2 the other virulence associated proteins should be investigated as potential vaccine candidates and these should be also be evaluated in heterologous prime boost regimes with various routes of vaccine administration require investigation including oral, nasal and intra peritoneal.

The results of this study show that the HAdV-*vapA* can potentially be developed further as an efficacious vaccine for *R*. *equi* vaccine. However, while this study is an important step in determining safety and efficacy of this novel vaccine candidate the trialling of this vaccine candidate in foals is crucial to determine if the HAdV-*vapA* vaccine candidate is effective in foals. Future studies will be designed to look at the safety and efficacy of this vaccine candidate in foals.
